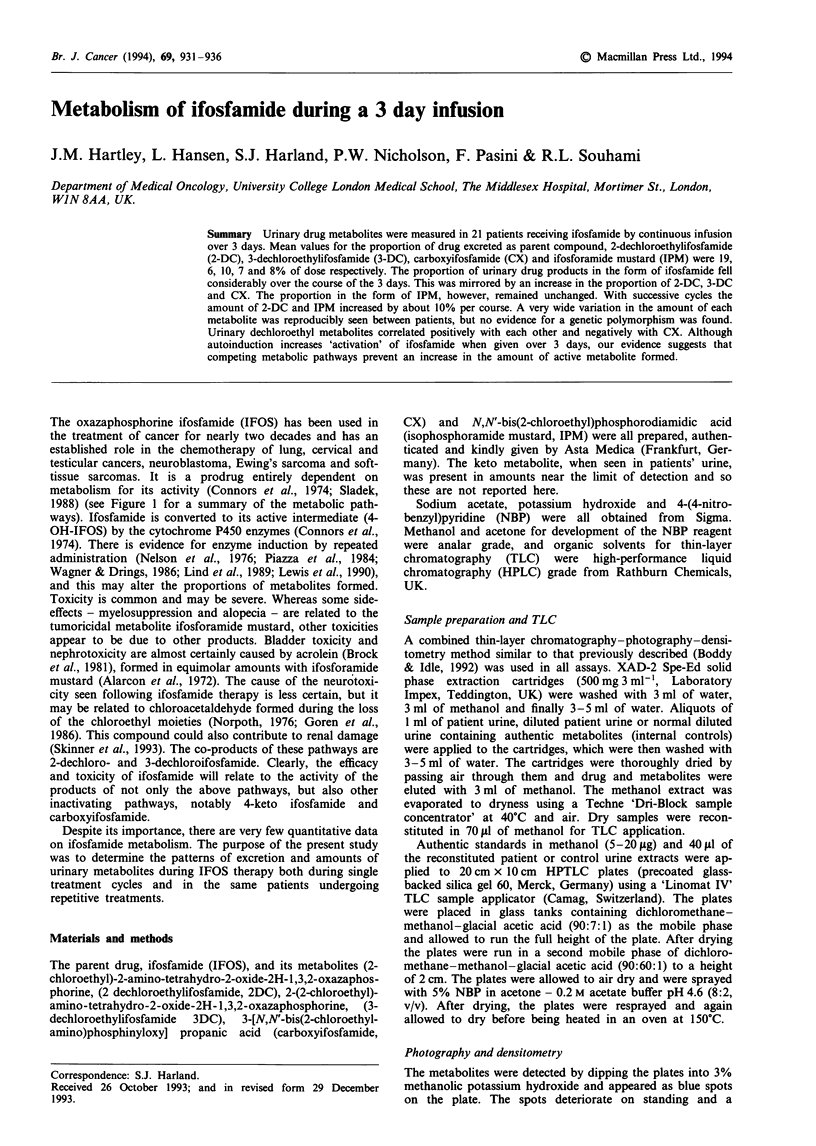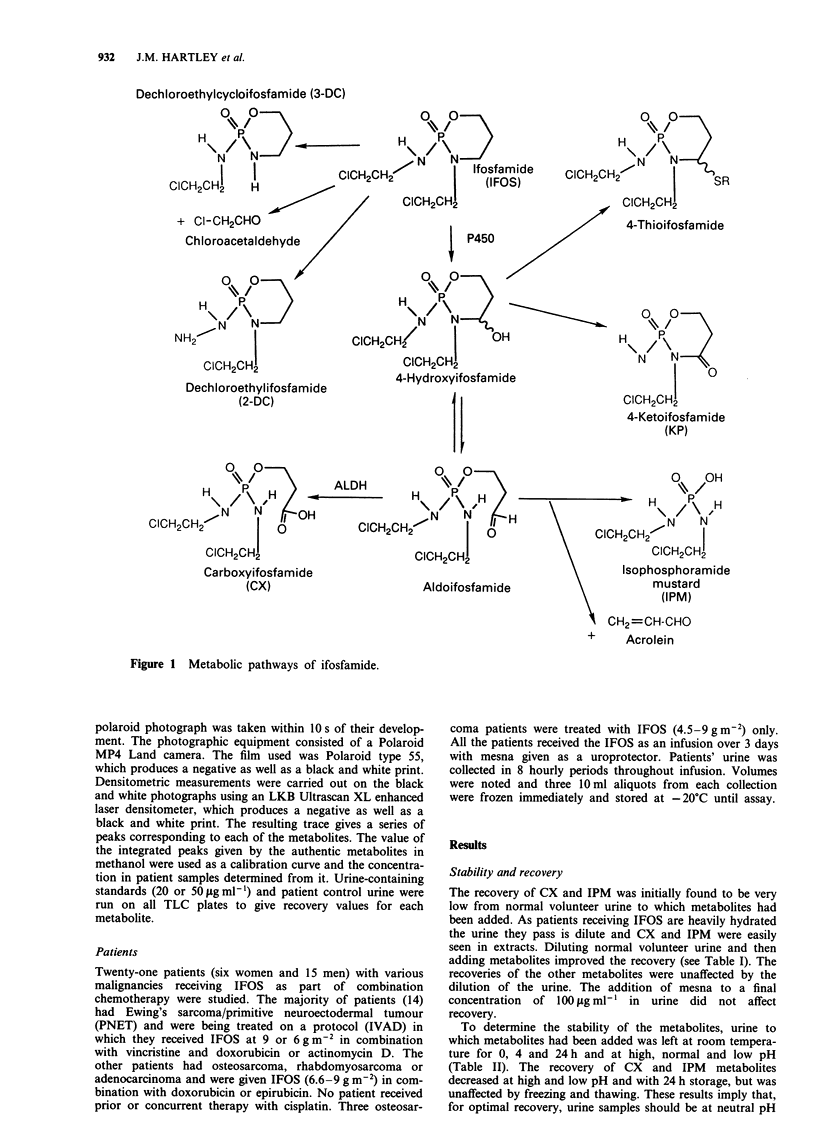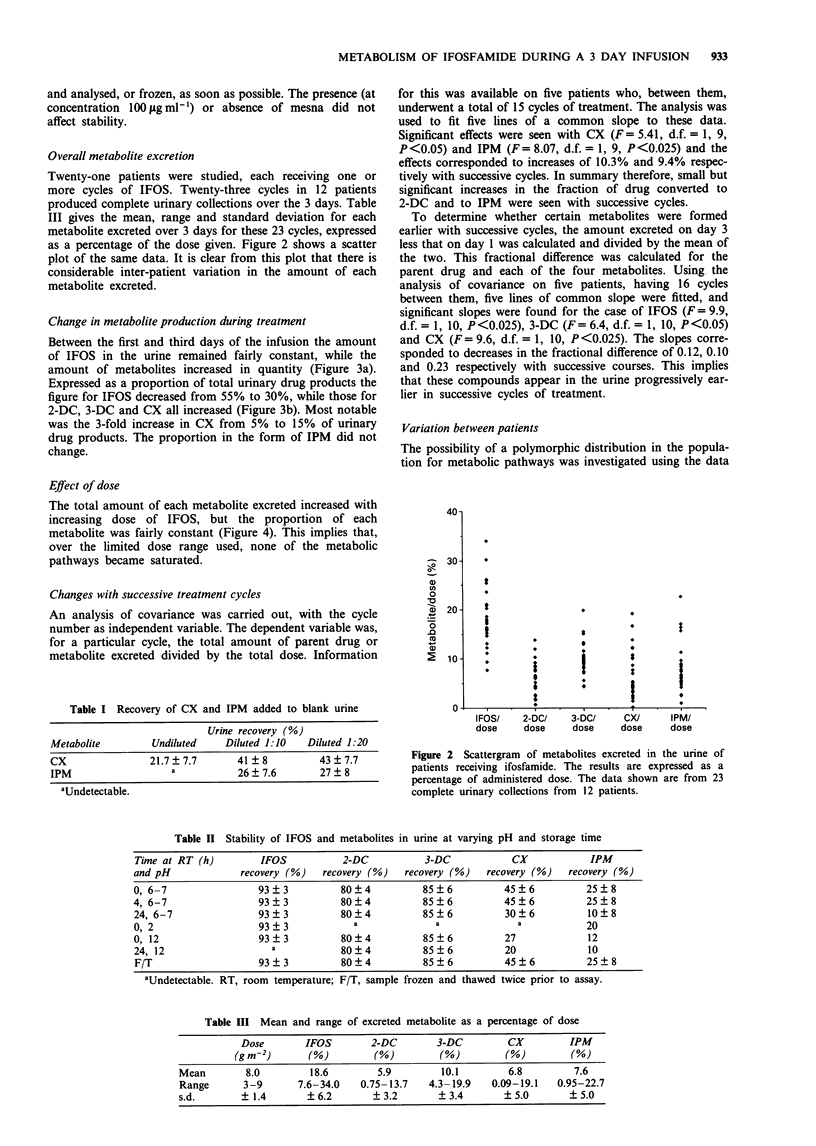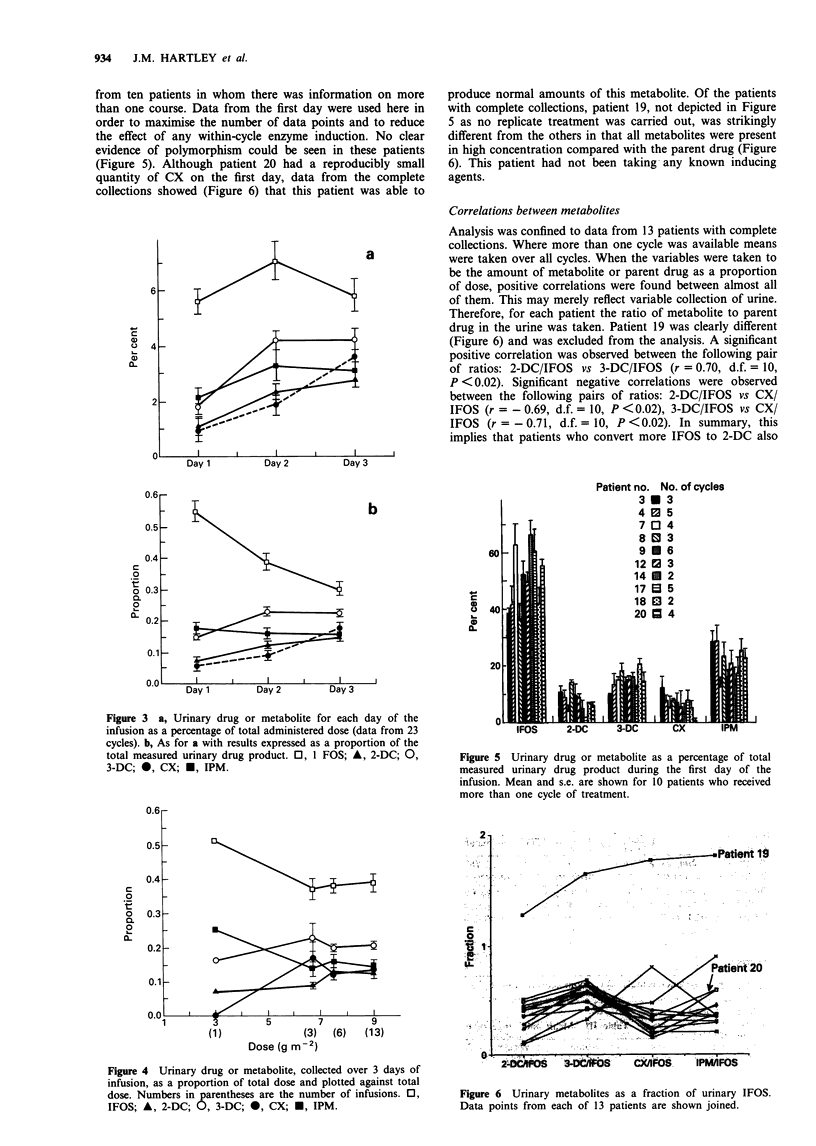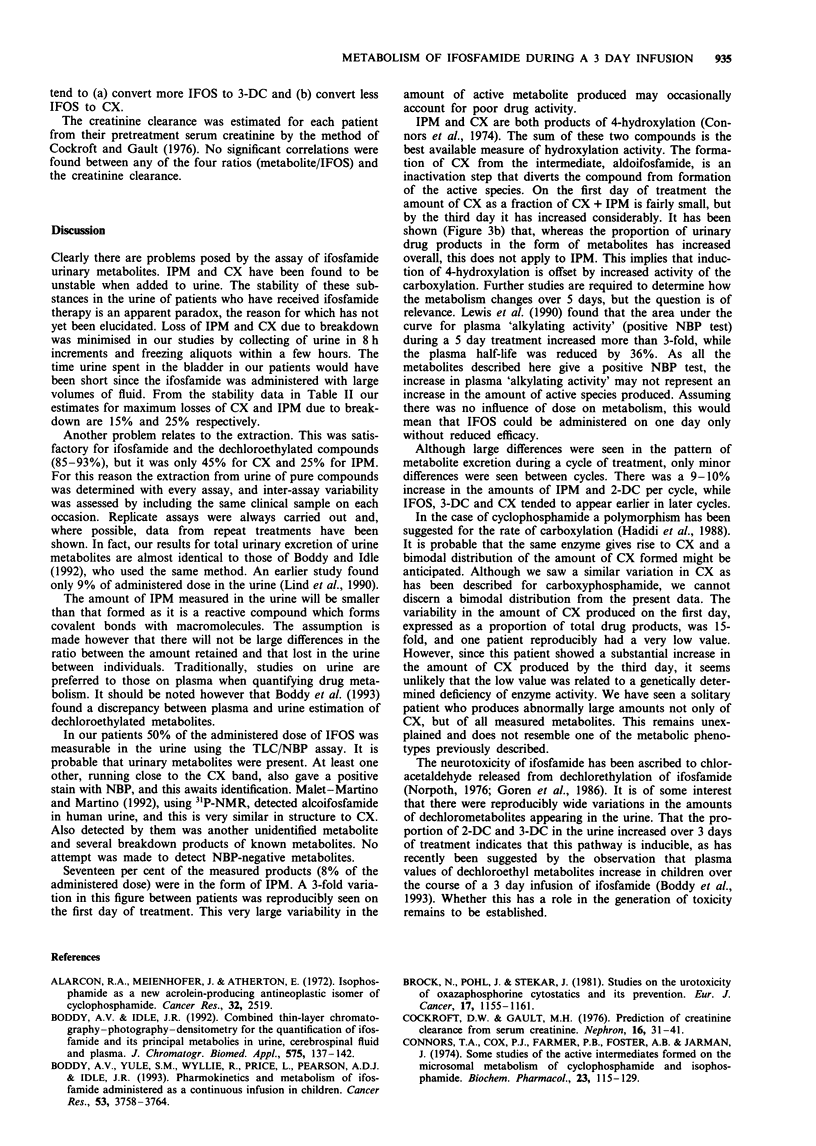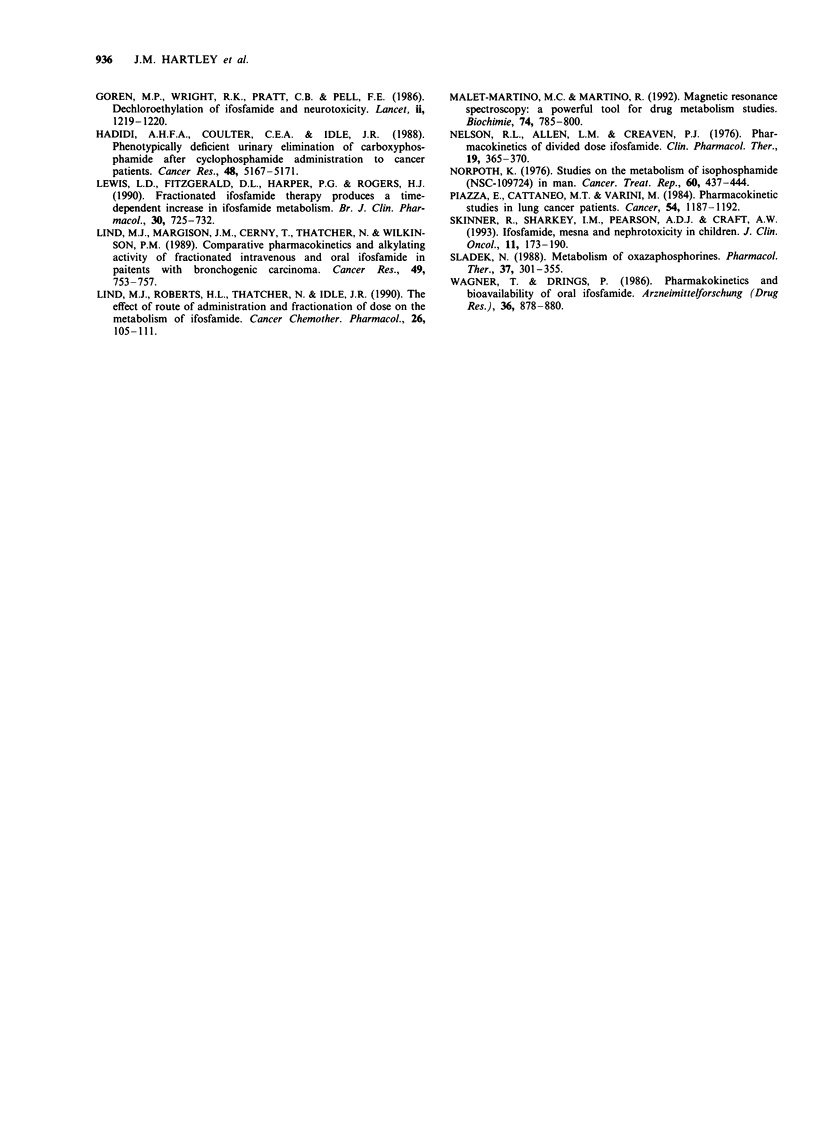# Metabolism of ifosfamide during a 3 day infusion.

**DOI:** 10.1038/bjc.1994.180

**Published:** 1994-05

**Authors:** J. M. Hartley, L. Hansen, S. J. Harland, P. W. Nicholson, F. Pasini, R. L. Souhami

**Affiliations:** Department of Medical Oncology, University College London Medical School, Middlesex Hospital, UK.

## Abstract

Urinary drug metabolites were measured in 21 patients receiving ifosfamide by continuous infusion over 3 days. Mean values for the proportion of drug excreted as parent compound, 2-dechloroethylifosfamide (2-DC), 3-dechloroethylifosfamide (3-DC), carboxyifosfamide (CX) and ifosforamide mustard (IPM) were 19, 6, 10, 7 and 8% of dose respectively. The proportion of urinary drug products in the form of ifosfamide fell considerably over the course of the 3 days. This was mirrored by an increase in the proportion of 2-DC, 3-DC and CX. The proportion in the form of IPM, however, remained unchanged. With successive cycles the amount of 2-DC and IPM increased by about 10% per course. A very wide variation in the amount of each metabolite was reproducibly seen between patients, but no evidence for a genetic polymorphism was found. Urinary dechloroethyl metabolites correlated positively with each other and negatively with CX. Although autoinduction increases 'activation' of ifosfamide when given over 3 days, our evidence suggests that competing metabolic pathways prevent an increase in the amount of active metabolite formed.


Br. J. Cancer (1994), 69, 931–936 © Macmillan Press Ltd., 1994

### Metabolism of ifosfamide during a 3 day infusion

J.M. Hartley, L. Hansen, S.J. Harland, P.W. Nicholson, F. Pasini & R.L. Souhami

*Department of Medical Oncology, University College London Medical School, The Middlesex Hospital, Mortimer St., London, W1N 8AA, UK.*

**Summary** Urinary drug metabolites were measured in 21 patients receiving ifosfamide by continuous infusion over 3 days. Mean values for the proportion of drug excreted as parent compound, 2-dechloroethylifosfamide (2-DC), 3-dechloroethylifosfamide (3-DC), carboxyifosfamide (CX) and ifosforamide mustard (IPM) were 19, 6, 10, 7 and 8% of dose respectively. The proportion of urinary drug products in the form of ifosfamide fell considerably over the course of the 3 days. This was mirrored by an increase in the proportion of 2-DC, 3-DC and CX. The proportion in the form of IPM, however, remained unchanged. With successive cycles the amount of 2-DC and IPM increased by about 10% per course. A very wide variation in the amount of each metabolite was reproducibly seen between patients, but no evidence for a genetic polymorphism was found. Urinary dechloroethyl metabolites correlated positively with each other and negatively with CX. Although autoinduction increases 'activation' of ifosfamide when given over 3 days, our evidence suggests that competing metabolic pathways prevent an increase in the amount of active metabolite formed.

The oxazaphosphorine ifosfamide (IFOS) has been used in the treatment of cancer for nearly two decades and has an established role in the chemotherapy of lung, cervical and testicular cancers, neuroblastoma, Ewing's sarcoma and soft-tissue sarcomas. It is a prodrug entirely dependent on metabolism for its activity (Connors *et al.*, 1974; Sladek, 1988) (see Figure 1 for a summary of the metabolic pathways). Ifosfamide is converted to its active intermediate (4-OH-IFOS) by the cytochrome P450 enzymes (Connors *et al.*, 1974). There is evidence for enzyme induction by repeated administration (Nelson *et al.*, 1976; Piazza *et al.*, 1984; Wagner & Drings, 1986; Lind *et al.*, 1989; Lewis *et al.*, 1990), and this may alter the proportions of metabolites formed. Toxicity is common and may be severe. Whereas some side-effects – myelosuppression and alopecia – are related to the tumoricidal metabolite ifosforamide mustard, other toxicities appear to be due to other products. Bladder toxicity and nephrotoxicity are almost certainly caused by acrolein (Brock *et al.*, 1981), formed in equimolar amounts with ifosforamide mustard (Alarcon *et al.*, 1972). The cause of the neurotoxicity seen following ifosfamide therapy is less certain, but it may be related to chloroacetaldehyde formed during the loss of the chloroethyl moieties (Norpoth, 1976; Goren *et al.*, 1986). This compound could also contribute to renal damage (Skinner *et al.*, 1993). The co-products of these pathways are 2-dechloro- and 3-dechloroifosfamide. Clearly, the efficacy and toxicity of ifosfamide will relate to the activity of the products of not only the above pathways, but also other inactivating pathways, notably 4-keto ifosfamide and carboxyifosfamide.

Despite its importance, there are very few quantitative data on ifosfamide metabolism. The purpose of the present study was to determine the patterns of excretion and amounts of urinary metabolites during IFOS therapy both during single treatment cycles and in the same patients undergoing repetitive treatments.

### Materials and methods

The parent drug, ifosfamide (IFOS), and its metabolites (2-chloroethyl)-2-amino-tetrahydro-2-oxide-2H-1,3,2-oxazaphosphorine, (2 dechloroethylifosfamide, 2DC), 2-(2-chloroethyl)-amino-tetrahydro-2-oxide-2H-1,3,2-oxazaphosphorine, (3-dechloroethylifosfamide 3DC), 3-[*N,N'*-bis(2-chloroethyl-amino)phosphinyloxy] propanic acid (carboxyifosfamide, CX) and *N,N'*-bis(2-chloroethyl)phosphorodiamidic acid (isophosphoramide mustard, IPM) were all prepared, authenticated and kindly given by Asta Medica (Frankfurt, Germany). The keto metabolite, when seen in patients' urine, was present in amounts near the limit of detection and so these are not reported here.

Sodium acetate, potassium hydroxide and 4-(4-nitrobenzyl)pyridine (NBP) were all obtained from Sigma. Methanol and acetone for development of the NBP reagent were analar grade, and organic solvents for thin-layer chromatography (TLC) were high-performance liquid chromatography (HPLC) grade from Rathburn Chemicals, UK.

### *Sample preparation and TLC*

A combined thin-layer chromatography–photography–densitometry method similar to that previously described (Boddy & Idle, 1992) was used in all assays. XAD-2 Spe-Ed solid phase extraction cartridges (500 mg 3 $ml^{-1}$, Laboratory Impex, Teddington, UK) were washed with 3 ml of water, 3 ml of methanol and finally 3–5 ml of water. Aliquots of 1 ml of patient urine, diluted patient urine or normal diluted urine containing authentic metabolites (internal controls) were applied to the cartridges, which were then washed with 3–5 ml of water. The cartridges were thoroughly dried by passing air through them and drug and metabolites were eluted with 3 ml of methanol. The methanol extract was evaporated to dryness using a Techne 'Dri-Block sample concentrator' at 40°C and air. Dry samples were reconstituted in 70 µl of methanol for TLC application.

Authentic standards in methanol (5–20 µg) and 40 µl of the reconstituted patient or control urine extracts were applied to 20 cm × 10 cm HPTLC plates (precoated glass-backed silica gel 60, Merck, Germany) using a 'Linomat IV' TLC sample applicator (Camag, Switzerland). The plates were placed in glass tanks containing dichloromethane–methanol–glacial acetic acid (90:7:1) as the mobile phase and allowed to run the full height of the plate. After drying the plates were run in a second mobile phase of dichloromethane–methanol–glacial acetic acid (90:60:1) to a height of 2 cm. The plates were allowed to air dry and were sprayed with 5% NBP in acetone – 0.2 M acetate buffer pH 4.6 (8:2, v/v). After drying, the plates were resprayed and again allowed to dry before being heated in an oven at 150°C.

### *Photography and densitometry*

The metabolites were detected by dipping the plates into 3% methanolic potassium hydroxide and appeared as blue spots on the plate. The spots deteriorate on standing and a

Correspondence: S.J. Harland.
Received 26 October 1993; and in revised form 29 December 1993.

**Figure 1** Metabolic pathways of ifosfamide.

polaroid photograph was taken within 10 s of their development. The photographic equipment consisted of a Polaroid MP4 Land camera. The film used was Polaroid type 55, which produces a negative as well as a black and white print. Densitometric measurements were carried out on the black and white photographs using an LKB Ultrascan XL enhanced laser densitometer, which produces a negative as well as a black and white print. The resulting trace gives a series of peaks corresponding to each of the metabolites. The value of the integrated peaks given by the authentic metabolites in methanol were used as a calibration curve and the concentration in patient samples determined from it. Urine-containing standards (20 or 50 $\mu g\ ml^{-1}$) and patient control urine were run on all TLC plates to give recovery values for each metabolite.

*Patients*

Twenty-one patients (six women and 15 men) with various malignancies receiving IFOS as part of combination chemotherapy were studied. The majority of patients (14) had Ewing's sarcoma/primitive neuroectodermal tumour (PNET) and were being treated on a protocol (IVAD) in which they received IFOS at 9 or 6 $g\ m^{-2}$ in combination with vincristine and doxorubicin or actinomycin D. The other patients had osteosarcoma, rhabdomyosarcoma or adenocarcinoma and were given IFOS (6.6–9 $g\ m^{-2}$) in combination with doxorubicin or epirubicin. No patient received prior or concurrent therapy with cisplatin. Three osteosarcoma patients were treated with IFOS (4.5–9 $g\ m^{-2}$) only. All the patients received the IFOS as an infusion over 3 days with mesna given as a uroprotector. Patients' urine was collected in 8 hourly periods throughout infusion. Volumes were noted and three 10 ml aliquots from each collection were frozen immediately and stored at −20°C until assay.

### Results

*Stability and recovery*

The recovery of CX and IPM was initially found to be very low from normal volunteer urine to which metabolites had been added. As patients receiving IFOS are heavily hydrated the urine they pass is dilute and CX and IPM were easily seen in extracts. Diluting normal volunteer urine and then adding metabolites improved the recovery (see Table I). The recoveries of the other metabolites were unaffected by the dilution of the urine. The addition of mesna to a final concentration of 100 $\mu g\ ml^{-1}$ in urine did not affect recovery.

To determine the stability of the metabolites, urine to which metabolites had been added was left at room temperature for 0, 4 and 24 h and at high, normal and low pH (Table II). The recovery of CX and IPM metabolites decreased at high and low pH and with 24 h storage, but was unaffected by freezing and thawing. These results imply that, for optimal recovery, urine samples should be at neutral pH

and analysed, or frozen, as soon as possible. The presence (at concentration 100 μg ml$^{-1}$) or absence of mesna did not affect stability.

*Overall metabolite excretion*

Twenty-one patients were studied, each receiving one or more cycles of IFOS. Twenty-three cycles in 12 patients produced complete urinary collections over the 3 days. Table III gives the mean, range and standard deviation for each metabolite excreted over 3 days for these 23 cycles, expressed as a percentage of the dose given. Figure 2 shows a scatter plot of the same data. It is clear from this plot that there is considerable inter-patient variation in the amount of each metabolite excreted.

*Change in metabolite production during treatment*

Between the first and third days of the infusion the amount of IFOS in the urine remained fairly constant, while the amount of metabolites increased in quantity (Figure 3a). Expressed as a proportion of total urinary drug products the figure for IFOS decreased from 55% to 30%, while those for 2-DC, 3-DC and CX all increased (Figure 3b). Most notable was the 3-fold increase in CX from 5% to 15% of urinary drug products. The proportion in the form of IPM did not change.

*Effect of dose*

The total amount of each metabolite excreted increased with increasing dose of IFOS, but the proportion of each metabolite was fairly constant (Figure 4). This implies that, over the limited dose range used, none of the metabolic pathways became saturated.

*Changes with successive treatment cycles*

An analysis of covariance was carried out, with the cycle number as independent variable. The dependent variable was, for a particular cycle, the total amount of parent drug or metabolite excreted divided by the total dose. Information for this was available on five patients who, between them, underwent a total of 15 cycles of treatment. The analysis was used to fit five lines of a common slope to these data. Significant effects were seen with CX ($F = 5.41$, d.f. = 1, 9, $P<0.05$) and IPM ($F = 8.07$, d.f. = 1, 9, $P<0.025$) and the effects corresponded to increases of 10.3% and 9.4% respectively with successive cycles. In summary therefore, small but significant increases in the fraction of drug converted to 2-DC and to IPM were seen with successive cycles.

To determine whether certain metabolites were formed earlier with successive cycles, the amount excreted on day 3 less that on day 1 was calculated and divided by the mean of the two. This fractional difference was calculated for the parent drug and each of the four metabolites. Using the analysis of covariance on five patients, having 16 cycles between them, five lines of common slope were fitted, and significant slopes were found for the case of IFOS ($F = 9.9$, d.f. = 1, 10, $P<0.025$), 3-DC ($F = 6.4$, d.f. = 1, 10, $P<0.05$) and CX ($F = 9.6$, d.f. = 1, 10, $P<0.025$). The slopes corresponded to decreases in the fractional difference of 0.12, 0.10 and 0.23 respectively with successive courses. This implies that these compounds appear in the urine progressively earlier in successive cycles of treatment.

*Variation between patients*

The possibility of a polymorphic distribution in the population for metabolic pathways was investigated using the data

**Table I** Recovery of CX and IPM added to blank urine

| Metabolite | Urine recovery (%) Undiluted | Diluted 1:10 | Diluted 1:20 |
|---|---|---|---|
| CX | 21.7 ± 7.7 | 41 ± 8 | 43 ± 7.7 |
| IPM | [a] | 26 ± 7.6 | 27 ± 8 |

[a]Undetectable.

**Figure 2** Scattergram of metabolites excreted in the urine of patients receiving ifosfamide. The results are expressed as a percentage of administered dose. The data shown are from 23 complete urinary collections from 12 patients.

**Table II** Stability of IFOS and metabolites in urine at varying pH and storage time

| Time at RT (h) and pH | IFOS recovery (%) | 2-DC recovery (%) | 3-DC recovery (%) | CX recovery (%) | IPM recovery (%) |
|---|---|---|---|---|---|
| 0, 6–7 | 93 ± 3 | 80 ± 4 | 85 ± 6 | 45 ± 6 | 25 ± 8 |
| 4, 6–7 | 93 ± 3 | 80 ± 4 | 85 ± 6 | 45 ± 6 | 25 ± 8 |
| 24, 6–7 | 93 ± 3 | 80 ± 4 | 85 ± 6 | 30 ± 6 | 10 ± 8 |
| 0, 2 | 93 ± 3 | [a] | [a] | [a] | 20 |
| 0, 12 | 93 ± 3 | 80 ± 4 | 85 ± 6 | 27 | 12 |
| 24, 12 | [a] | 80 ± 4 | 85 ± 6 | 20 | 10 |
| F/T | 93 ± 3 | 80 ± 4 | 85 ± 6 | 45 ± 6 | 25 ± 8 |

[a]Undetectable. RT, room temperature; F/T, sample frozen and thawed twice prior to assay.

**Table III** Mean and range of excreted metabolite as a percentage of dose

| | Dose (g m$^{-2}$) | IFOS (%) | 2-DC (%) | 3-DC (%) | CX (%) | IPM (%) |
|---|---|---|---|---|---|---|
| Mean | 8.0 | 18.6 | 5.9 | 10.1 | 6.8 | 7.6 |
| Range | 3–9 | 7.6–34.0 | 0.75–13.7 | 4.3–19.9 | 0.09–19.1 | 0.95–22.7 |
| s.d. | ± 1.4 | ± 6.2 | ± 3.2 | ± 3.4 | ± 5.0 | ± 5.0 |

from ten patients in whom there was information on more than one course. Data from the first day were used here in order to maximise the number of data points and to reduce the effect of any within-cycle enzyme induction. No clear evidence of polymorphism could be seen in these patients (Figure 5). Although patient 20 had a reproducibly small quantity of CX on the first day, data from the complete collections showed (Figure 6) that this patient was able to produce normal amounts of this metabolite. Of the patients with complete collections, patient 19, not depicted in Figure 5 as no replicate treatment was carried out, was strikingly different from the others in that all metabolites were present in high concentration compared with the parent drug (Figure 6). This patient had not been taking any known inducing agents.

**Figure 3** **a,** Urinary drug or metabolite for each day of the infusion as a percentage of total administered dose (data from 23 cycles). **b,** As for **a** with results expressed as a proportion of the total measured urinary drug product. □, 1 FOS; ▲, 2-DC; ○, 3-DC; ●, CX; ■, IPM.

**Figure 4** Urinary drug or metabolite, collected over 3 days of infusion, as a proportion of total dose and plotted against total dose. Numbers in parentheses are the number of infusions. □, IFOS; ▲, 2-DC; ○, 3-DC; ●, CX; ■, IPM.

### *Correlations between metabolites*

Analysis was confined to data from 13 patients with complete collections. Where more than one cycle was available means were taken over all cycles. When the variables were taken to be the amount of metabolite or parent drug as a proportion of dose, positive correlations were found between almost all of them. This may merely reflect variable collection of urine. Therefore, for each patient the ratio of metabolite to parent drug in the urine was taken. Patient 19 was clearly different (Figure 6) and was excluded from the analysis. A significant positive correlation was observed between the following pair of ratios: 2-DC/IFOS *vs* 3-DC/IFOS ($r = 0.70$, d.f. = 10, $P < 0.02$). Significant negative correlations were observed between the following pairs of ratios: 2-DC/IFOS *vs* CX/IFOS ($r = -0.69$, d.f. = 10, $P < 0.02$), 3-DC/IFOS *vs* CX/IFOS ($r = -0.71$, d.f. = 10, $P < 0.02$). In summary, this implies that patients who convert more IFOS to 2-DC also

**Figure 5** Urinary drug or metabolite as a percentage of total measured urinary drug product during the first day of the infusion. Mean and s.e. are shown for 10 patients who received more than one cycle of treatment.

**Figure 6** Urinary metabolites as a fraction of urinary IFOS. Data points from each of 13 patients are shown joined.

tend to (a) convert more IFOS to 3-DC and (b) convert less IFOS to CX.

The creatinine clearance was estimated for each patient from their pretreatment serum creatinine by the method of Cockroft and Gault (1976). No significant correlations were found between any of the four ratios (metabolite/IFOS) and the creatinine clearance.

### Discussion

Clearly there are problems posed by the assay of ifosfamide urinary metabolites. IPM and CX have been found to be unstable when added to urine. The stability of these substances in the urine of patients who have received ifosfamide therapy is an apparent paradox, the reason for which has not yet been elucidated. Loss of IPM and CX due to breakdown was minimised in our studies by collecting of urine in 8 h increments and freezing aliquots within a few hours. The time urine spent in the bladder in our patients would have been short since the ifosfamide was administered with large volumes of fluid. From the stability data in Table II our estimates for maximum losses of CX and IPM due to breakdown are 15% and 25% respectively.

Another problem relates to the extraction. This was satisfactory for ifosfamide and the dechloroethylated compounds (85–93%), but it was only 45% for CX and 25% for IPM. For this reason the extraction from urine of pure compounds was determined with every assay, and inter-assay variability was assessed by including the same clinical sample on each occasion. Replicate assays were always carried out and, where possible, data from repeat treatments have been shown. In fact, our results for total urinary excretion of urine metabolites are almost identical to those of Boddy and Idle (1992), who used the same method. An earlier study found only 9% of administered dose in the urine (Lind *et al.*, 1990).

The amount of IPM measured in the urine will be smaller than that formed as it is a reactive compound which forms covalent bonds with macromolecules. The assumption is made however that there will not be large differences in the ratio between the amount retained and that lost in the urine between individuals. Traditionally, studies on urine are preferred to those on plasma when quantifying drug metabolism. It should be noted however that Boddy *et al.* (1993) found a discrepancy between plasma and urine estimation of dechloroethylated metabolites.

In our patients 50% of the administered dose of IFOS was measurable in the urine using the TLC/NBP assay. It is probable that urinary metabolites were present. At least one other, running close to the CX band, also gave a positive stain with NBP, and this awaits identification. Malet–Martino and Martino (1992), using $^{31}$P-NMR, detected alcoifosfamide in human urine, and this is very similar in structure to CX. Also detected by them was another unidentified metabolite and several breakdown products of known metabolites. No attempt was made to detect NBP-negative metabolites.

Seventeen per cent of the measured products (8% of the administered dose) were in the form of IPM. A 3-fold variation in this figure between patients was reproducibly seen on the first day of treatment. This very large variability in the amount of active metabolite produced may occasionally account for poor drug activity.

IPM and CX are both products of 4-hydroxylation (Connors *et al.*, 1974). The sum of these two compounds is the best available measure of hydroxylation activity. The formation of CX from the intermediate, aldoifosfamide, is an inactivation step that diverts the compound from formation of the active species. On the first day of treatment the amount of CX as a fraction of CX + IPM is fairly small, but by the third day it has increased considerably. It has been shown (Figure 3b) that, whereas the proportion of urinary drug products in the form of metabolites has increased overall, this does not apply to IPM. This implies that induction of 4-hydroxylation is offset by increased activity of the carboxylation. Further studies are required to determine how the metabolism changes over 5 days, but the question is of relevance. Lewis *et al.* (1990) found that the area under the curve for plasma 'alkylating activity' (positive NBP test) during a 5 day treatment increased more than 3-fold, while the plasma half-life was reduced by 36%. As all the metabolites described here give a positive NBP test, the increase in plasma 'alkylating activity' may not represent an increase in the amount of active species produced. Assuming there was no influence of dose on metabolism, this would mean that IFOS could be administered on one day only without reduced efficacy.

Although large differences were seen in the pattern of metabolite excretion during a cycle of treatment, only minor differences were seen between cycles. There was a 9–10% increase in the amounts of IPM and 2-DC per cycle, while IFOS, 3-DC and CX tended to appear earlier in later cycles.

In the case of cyclophosphamide a polymorphism has been suggested for the rate of carboxylation (Hadidi *et al.*, 1988). It is probable that the same enzyme gives rise to CX and a bimodal distribution of the amount of CX formed might be anticipated. Although we saw a similar variation in CX as has been described for carboxyphosphamide, we cannot discern a bimodal distribution from the present data. The variability in the amount of CX produced on the first day, expressed as a proportion of total drug products, was 15-fold, and one patient reproducibly had a very low value. However, since this patient showed a substantial increase in the amount of CX produced by the third day, it seems unlikely that the low value was related to a genetically determined deficiency of enzyme activity. We have seen a solitary patient who produces abnormally large amounts not only of CX, but of all measured metabolites. This remains unexplained and does not resemble one of the metabolic phenotypes previously described.

The neurotoxicity of ifosfamide has been ascribed to chloracetaldehyde released from dechlorethylation of ifosfamide (Norpoth, 1976; Goren *et al.*, 1986). It is of some interest that there were reproducibly wide variations in the amounts of dechlorometabolites appearing in the urine. That the proportion of 2-DC and 3-DC in the urine increased over 3 days of treatment indicates that this pathway is inducible, as has recently been suggested by the observation that plasma values of dechloroethyl metabolites increase in children over the course of a 3 day infusion of ifosfamide (Boddy *et al.*, 1993). Whether this has a role in the generation of toxicity remains to be established.

### References

ALARCON, R.A., MEIENHOFER, J. & ATHERTON, E. (1972). Isophosphamide as a new acrolein-producing antineoplastic isomer of cyclophosphamide. *Cancer Res.*, **32**, 2519.

BODDY, A.V. & IDLE, J.R. (1992). Combined thin-layer chromatography–photography–densitometry for the quantification of ifosfamide and its principal metabolies in urine, cerebrospinal fluid and plasma. *J. Chromatogr. Biomed. Appl.*, **575**, 137–142.

BODDY, A.V., YULE, S.M., WYLLIE, R., PRICE, L., PEARSON, A.D.J. & IDLE, J.R. (1993). Pharmokinetics and metabolism of ifosfamide administered as a continuous infusion in children. *Cancer Res.*, **53**, 3758–3764.

BROCK, N., POHL, J. & STEKAR, J. (1981). Studies on the urotoxicity of oxazaphosphorine cytostatics and its prevention. *Eur. J. Cancer*, **17**, 1155–1161.

COCKROFT, D.W. & GAULT, M.H. (1976). Prediction of creatinine clearance from serum creatinine. *Nephron*, **16**, 31–41.

CONNORS, T.A., COX, P.J., FARMER, P.B., FOSTER, A.B. & JARMAN, J. (1974). Some studies of the active intermediates formed on the microsomal metabolism of cyclophosphamide and isophosphamide. *Biochem. Pharmacol.*, **23**, 115–129.

GOREN, M.P., WRIGHT, R.K., PRATT, C.B. & PELL, F.E. (1986). Dechloroethylation of ifosfamide and neurotoxicity. *Lancet*, **ii**, 1219–1220.

HADIDI, A.H.F.A., COULTER, C.E.A. & IDLE, J.R. (1988). Phenotypically deficient urinary elimination of carboxyphosphamide after cyclophosphamide administration to cancer patients. *Cancer Res.*, **48**, 5167–5171.

LEWIS, L.D., FITZGERALD, D.L., HARPER, P.G. & ROGERS, H.J. (1990). Fractionated ifosfamide therapy produces a time-dependent increase in ifosfamide metabolism. *Br. J. Clin. Pharmacol.*, **30**, 725–732.

LIND, M.J., MARGISON, J.M., CERNY, T., THATCHER, N. & WILKINSON, P.M. (1989). Comparative pharmacokinetics and alkylating activity of fractionated intravenous and oral ifosfamide in paitents with bronchogenic carcinoma. *Cancer Res.*, **49**, 753–757.

LIND, M.J., ROBERTS, H.L., THATCHER, N. & IDLE, J.R. (1990). The effect of route of administration and fractionation of dose on the metabolism of ifosfamide. *Cancer Chemother. Pharmacol.*, **26**, 105–111.

MALET-MARTINO, M.C. & MARTINO, R. (1992). Magnetic resonance spectroscopy: a powerful tool for drug metabolism studies. *Biochimie*, **74**, 785–800.

NELSON, R.L., ALLEN, L.M. & CREAVEN, P.J. (1976). Pharmacokinetics of divided dose ifosfamide. *Clin. Pharmacol. Ther.*, **19**, 365–370.

NORPOTH, K. (1976). Studies on the metabolism of isophosphamide (NSC-109724) in man. *Cancer. Treat. Rep.*, **60**, 437–444.

PIAZZA, E., CATTANEO, M.T. & VARINI, M. (1984). Pharmacokinetic studies in lung cancer patients. *Cancer*, **54**, 1187–1192.

SKINNER, R., SHARKEY, I.M., PEARSON, A.D.J. & CRAFT, A.W. (1993). Ifosfamide, mesna and nephrotoxicity in children. *J. Clin. Oncol.*, **11**, 173–190.

SLADEK, N. (1988). Metabolism of oxazaphosphorines. *Pharmacol. Ther.*, **37**, 301–355.

WAGNER, T. & DRINGS, P. (1986). Pharmakokinetics and bioavailability of oral ifosfamide. *Arzneimittelforschung (Drug Res.)*, **36**, 878–880.